# Direct Synthesis of Fe-Al Alloys from Elemental Powders Using Laser Engineered Net Shaping

**DOI:** 10.3390/ma13030531

**Published:** 2020-01-22

**Authors:** Magda Pęska, Krzysztof Karczewski, Magdalena Rzeszotarska, Marek Polański

**Affiliations:** Institute of Materials Science and Engineering, Military University of Technology, gen. S. Kaliskiego 2 St., 00-908 Warsaw, Poland; magda.peska@wat.edu.pl (M.P.); magdalena.rzeszotarska@wat.edu.pl (M.R.); marek.polanski@wat.edu.pl (M.P.)

**Keywords:** intermetallics, FeAl, Fe_3_Al, additive manufacturing, LENS, synthesis

## Abstract

The laser engineered net shaping (LENS®) process is shown here as an alternative to melting, casting, and powder metallurgy for manufacturing iron aluminides. This technique was found to allow for the production of FeAl and Fe_3_Al phases from mixtures of elemental iron and aluminum powders. The in situ synthesis reduces the manufacturing cost and enhances the manufacturing efficiency due to the control of the chemical and phase composition of the deposited layers. The research was carried out on samples with different chemical compositions that were deposited on the intermetallic substrates that were produced by powder metallurgy. The obtained samples with the desired phase composition illustrated that LENS® technology can be successfully applied to alloys synthesis.

## 1. Introduction

Fe-Al intermetallic compounds have attracted the attention of many researchers due to their unique properties, including oxidation and corrosion resistance [1,2,3,4,5,6], considerable high-temperature strength, and creep resistance [7,8,9,10,11]. In addition, a significant advantage of Fe-Al alloys is the low cost of substrates, which enables an economically justified manufacturing process [12,13,14,15,16]. However, the widespread use of Fe-Al intermetallic compounds is limited, mainly due to their low ductility at room temperature [10,17,18,19]. However, it is worth to note that, under properly chosen conditions (deformation speed and temperature), those alloys can undergo significant deformation [20,21,22,23,24] and they were even subjected to successful severe plastic deformation processes [25,26]. Fe_3_Al and FeAl are the phases that are most frequently considered for industrial applications, because both have an A2-derived structure that is usually related to reasonably acceptable ductility. According to the binary phase diagram, FeAl has a B2 crystal structure, which occurs in the range of 36–50 at.% Al at room temperature and it is stable up to the melting point. Fe_3_Al occurs in the 23–36 at.% Al range and might possess two crystal structures: B2 and D03. At temperature above 550 °C, Fe_3_Al transforms from the D03 crystal structure to a cubic B2-type structure [27,28,29].

The effective use of these materials involves finding the proper fabrication technique, without losing the benefit of the low cost [30]. These alloys can be obtained by various methods, such as conventional melting and casting or powder metallurgy (using elemental powders or pre-alloyed powders), with the engagement of so-called SHS (Self-propagating High-temperature Synthesis) [31,32,33,34]. 

Induction melting is considered as one of the most economical melting processes. Moreover, it is widely used in mass production of alloys with non-reactive components. However, this method cannot be easily used for the mass production of FeAl-based alloys. Attempts at induction melting in the air atmosphere lead to very high gas porosity of the obtained materials. This porosity results from the reaction between water vapor from the air and highly reactive aluminum in liquid metal [30]. The use of electroslag re-melting is another method of casting. Free of gas porosity ingots were obtained with this approach. R. G. Baligidad et al. [35], conducted research on Fe_3_Al alloy fabrication with this method but found the problem with determining the appropriate process parameters. Another type of melting and casting method is the Exo-Melt™ process, which uses the heat of the exothermic reaction that occurs between aluminum and iron during the formation of the FeAl or Fe_3_Al phase. The process is carried out in an induction furnace, where the components are specially arranged over the furnace volume, thus the heat accompanying the exothermic reaction supports melting. The Exo-Melt™ process is commercially used by United Defense LP (Anniston, AL, USA) and Alloy Engineering and Casting Company (Champaign, IL, USA) [36]. 

The powder metallurgy process allows for obtaining finished products with complex shapes. It also provides much easier control of the materials’ structure (including grain size), when compared to the traditional melting and casting [37]. Pre-alloyed powders can be used as a starter material (FeAl or Fe_3_Al) for iron aluminides manufacturing. However, hot pressing (HP) or hot isostatic pressing (HIP) methods are usually usedto improve the final properties of the product due to their fairly high melting point and hardness. Unfortunately, this involves the use of complex procedures, which raise the costs of the entire process and reduces its efficiency. Therefore, the industrial-scale application of this type method faces certain limitations [38]. Nevertheless, there is a certain by-pass: in the powder metallurgy of the Fe-Al alloys, it is possible to use elemental powder mixtures, while employing a two-stage SHS reaction, which occurs during these process, as already mentioned [31,32,33,34]. The reaction allows for obtaining samples with the assumed chemical composition, but phase structure is not usually easy to handle without long-lasting annealing and is strongly dependant on the heating rate, as it follows several stages usually before obtaining the equilibrium [39,40,41]. The main disadvantage of powder metallurgy is that materials obtained by this reaction usually possess porosity, which can sometimes reach up high values, sometimes even intentionally [42,43,44].

These materials have also been produced by additive manufacturing technology, such as selective laser melting (SLM) [45] or laser engineered net shaping (LENS®), according to recent works [46].

The amount of research in additive manufacturing has significantly increased over the past few years [47], because this group of techniquesopens unique manufacturing opportunities in terms of geometry [48] and the complexity of manufactured structures. Another significant advantage of additive manufacturing is (at least theoretically) the effective use of the applied powder material. The un-melted powder can usually be sieved and reused; it is important to note that this is not the case when more than one type of powder is used during the process. In contrast, in subtractive manufacturing, the material removed during fabrication forms waste-like turnings [49,50]. In most additive manufacturing techniques, items are manufactured layer-by-layer while using thermal energy (of different origin e.g. laser, electron beam, kinetic energy) to add new layers [51]. Moreover, the parts can be made of a variety of materials, such as plastics, composites, or metals [52].

Laser Engineered Net Shaping (LENS®) was employed in our recent research. Most of the industrially used additive manufacturing methods that are based on the so-called powder-bed conception. In this kind of route, metallic powder is being spread over the working surface and the requested shape is then remelted with the use of very focused (tens of microns) and very fast-moving (meter per second) laser beam. The sintered or melted layer is then lowered and covered by the next layer of “fresh powder”, which is then remelted. In such a case, the final product “grows” in the powder bed. These kinds of methods are providing the best surface quality and great precision. However, the methods themselves do not allow for making any kind of differentiation of chemical composition over the sample volume (except some rare cases of the gradient in Z-axis).

LENS is an additive manufacturing process, from so-called DED (Direct Energy Deposition) group, where metal (or sometimes ceramic) powders are used to build various structural and functional parts while being blown directly to the molten metal pool formed by the (usually) laser beam. As the powders are supplied from external powder feeders, which can contain different alloys, they can be mixed at different ratios and introduced to the molten pool at different proportions. In such case, the chemical composition of manufactured components can change within X,Y (usually) and Z (usually) axes. Cladding on the surface, joining of two different metals, building gradient structures is available. It is worth to note that commercially available powders, dedicated to additive manufacturing, are quite expensive, due to the specific requirements, such as proper shape or size of powder particles. Thus far, research has been carried out to develop metal powders that would best fulfill application requirements while being economical [53].

Usually, the pre-alloyed powders with the desired, tailored for further applications, chemical composition are used in LENS®. However, due to the fact that the LENS® system uses powder feedstock that is theoretically possible to blend the elemental powders, it could potentially reduce the costs and, while considering that the system is equipped with many nozzles, allows for the creation of gradient materials. 

There are known attempts of manufacturing the alloys (also intermetallics by additive manufacturing), where elemental blends were successfully used and their behavior was correlated with the enthalpy of mixing [54,55,56,57,58,59]. Banerjee et al. also performed attempts of making composite materials [60]. Significant differences were noticed for alloys made of elemental powder with positive and negative enthalpies of mixing.

Despite the big difficulty, trials of application of SLM technique for alloying from elemental powders were also conducted.Grigoriev and Polozov managed to synthesize titanium alloys [61,62]. Additionally, Nazarov et al. took an attempt to fabricate Ni-Al alloys [63]. A very special combination of additive manufacturing, together with the utilization of SHS reaction was also applied by Shiskovskii et al. [64,65]. 

There is only a limited number of studies focused on the deposition of alloys with LENS® or similar techniques while using elemental powders and the work is mostly focused on pre-alloyed powders while considering the huge amount of alloys and intermetallics alloys proposed to be used [66,67,68,69,70,71]. The results of these experiments suggested that the enthalpy of mixing of the elements is one of the most important factors determining the heterogeneity and microstructure of the obtained materials. The use of mixtures of elemental powders that are characterized by endothermic or weakly exothermic mixing and, additionally, with the high melting point will require the use of much higher laser beam energy. Elements that mix exothermically provide an additional heat into the liquid melt pool, acting as an additional source of energy, which affects the possibility of using less laser beam energy, as well as reducing the critical energy density that is necessary for achieving chemical homogeneity [59]. However, the reaction is not easy to control, which makes the process more difficult to conduct and to obtain the desired shapes of the samples.

Therefore, the main purpose of the presented experiment was to determine the possibility of direct synthesis of Fe-Al alloys that were obtained by the LENS® technique, while using mixtures of elementary powders. The attempt of FeAl alloys direct energy deposition was already taken by Shiskovskii et al. [72]. Authors show the great potential of this attitude to fabricate even alloys with high aluminum content; however, in their experiment, the aluminum alloy powder was used instead of pure aluminum, which made the experiment more practical, but many variables were introduced in this case and it is quite hard to translate this results to a pure two-component system. 

The use of a completely different technique might prove to be an appropriate solution for manufacturing these intermetallic alloys, for example, as coatings for complex geometry surfaces due to already mentioned difficulties in obtaining Fe-Al materials by conventional methods. There have been studies comparing the mechanical properties of the same materials that were obtained while using the LENS® technique and using the other methods. Interestingly, the results of these studies clearly showed that the parts produced in the LENS® process were characterized by better mechanical properties due to the fine microstructure resulting from specific cooling conditions and high cooling rates [73]. 

## 2. Methods

### 2.1. Samples Manufacturing

**Figure 1:**
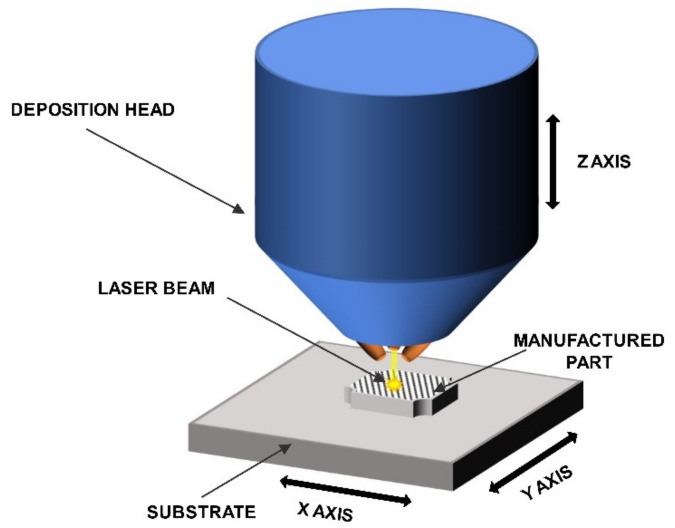
Schematic of the LENS® process.

The LENS® process was used to manufacture the intermetallic samples. This DED-type (direct energy deposition) process enables the layer by layer formation of fully dense and functional parts while using powders or powders mixtures as a starting material. The machine that was used in this particular experiment was custom made on the base of MR-7 (Optomec, Albuquerque, NM, USA) and it provides the opportunity to compose various powder mixtures delivered from up to four powder feeders at the same time. The software and hardware is configured in the way that multi-material samples, even sample libraries and gradient materials [67,74,75,76], can be built while using elemental powders or premixed powders up to user’s choice.This feature provides the most significant difference between LENS process and the most widely used powder bed type methods, where the layer of the powder is spread over the table, then remelted, lowered, and the process is repeated. The system is equipped with a fiber laser with a maximum power of 500W and a thermal imager system for an investigation of the cooling rates withing the molten metal pool (if necessary). The powder is delivered in argon stream by a system of four nozzles. The nozzles were designed, so that the powder streams converge at the same point as the focused laser beam. The whole process is carried out in an argon atmosphere (5N minimum) in a closed, constantly purified glovebox. The samples are being built on the movable table (in X and Y axes) that moves relative to the laser beam. The control is performed with a computer to produce layers with designed shapes. Figure 1 shows a schematic of the LENS® process.

Intermetallic sinters were prepared and used as a substrate to deposit on.in order to reduce the chemical incompatibility between the deposited material and the substrate. Iron and aluminum powders were mixed in relevant proportions and then initial uniaxial-load od 500 MPa was applied to form a molding with 50 mm diameter and about 10 mm in height. Subsequently, each sample was transferred to a graphite die and then sintered in a vacuum under 25MPa static uniaxial load for 15 minutes at the temperature of 1050 °C. The substrate plates were then annealed for 40 h at 1000 °C, ground with the use of a surface grinder, and sandblasted to avoid a shiny surface. 

### 2.2. Samples Characterization

Particle size is an important factor and powders that were within the range of 40μm to 150 μm should be used to ensure the proper flow of the powder. Thus, particle size analysis was carried out with a Kamika IPS UA particle analyzer (KAMIKA Instruments, Warsaw, Poland).

Structure analysis was carried out on an optical microscope (Nikon MA 200, Nikon Corporation, Tokyo, Japan). Scanning electron microscopy (Philips XL30/LaB6, Philips, Eindhoven, The Netherlands) was used for morphology observations (secondary electron mode). The tests were carried out with an SEM-EDAX X-ray microprobe (AMETEK, Inc., New Jersey, USA), and the chemical composition was determined by analyzing the radiation energy spectrum. During the study, ZAF correlations were used, so that the results of the measurements were not influenced by absorption or fluorescence.

The X-ray diffraction patterns (XRD) tests were carried out with a Rigaku ULTIMA IV diffractometer (Rigaku Corporation, Tokyo, Japan) using CoKα radiation in a2θ range of 20–140° with a scanning step of 0.02° and a scanning speed of 1°/min. The results were analyzed while using the DHN PDF 4 crystallographic database and PDXL (Rigaku) software (version 2.8.4.0).

Microhardness tests were performed with the use of SHIMADZU HMV-G series hardness tester (Shimadzu Corporation, Kyoto, Japan) while using the Vickers indenter and a load of 200g.

## 3. Materials and Preliminary Research

**Figure 2:**
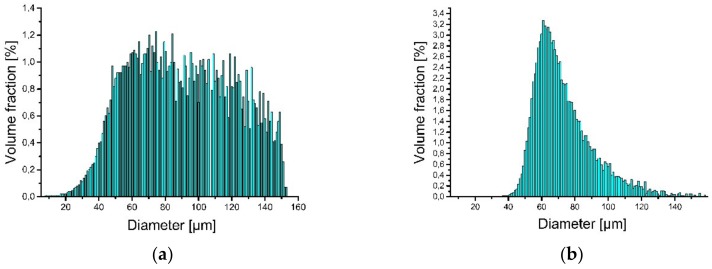
Particle size distribution of powder used to produce LENS® samples: (**a**) iron and (**b**) aluminum.

Iron (supplied by TLS Technik GmbH & Co. Spezialpulver, Bitterfeld-Wolfen, Germany)and aluminum (LPW Technology, Widnes, UK) elemental powders were used as the starting materials for the LENS® processes. Particle size analysis revealed that approximately 92% of the iron and approximately 93% of the aluminum powder belongs to the desired granulometric range (Figure 2).

**Figure 3:**
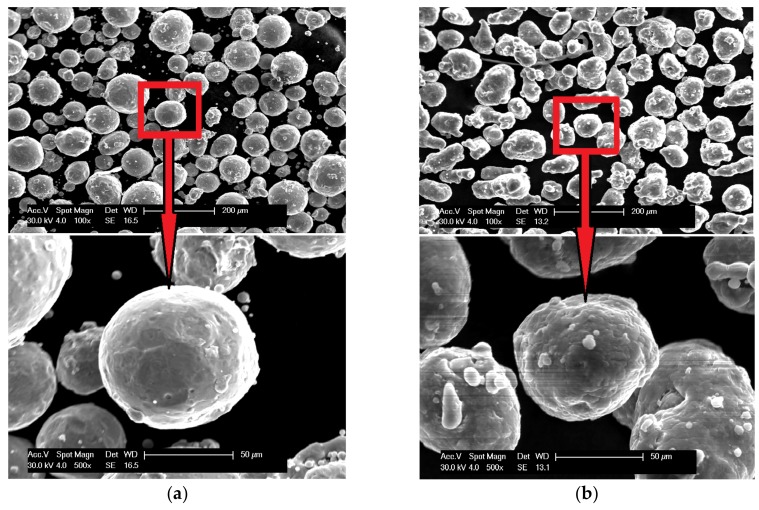
Scanning electron micrographs of powder morphology:(**a**) iron and (**b**) aluminum.

**Figure 4:**
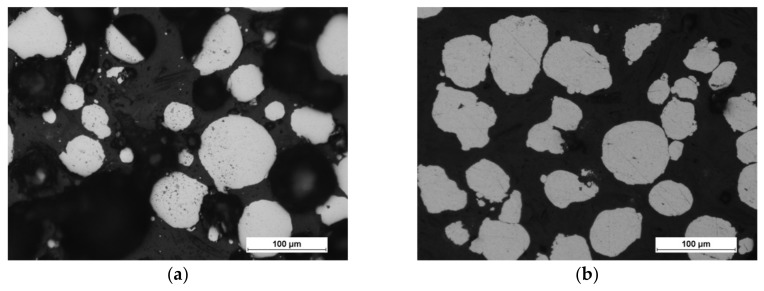
Optical micrographs of powder morphology: (**a**) iron and (**b**) aluminum.

The scanning electron microscopy observations were carried out and confirmed the spherical shape of both powders (aluminum particles are more elongated, but still close to spherical), and Figure 3 shows the results. The powders (bound with the thermoset resin, ground and polished) were subjected to the shape analysis with the use of the NIS (Nikon) software (version 3.2 BR). The results show the shape factor of α_Al_=0,62 ± 0,21, and α_Fe_=0,74 ± 0,21for aluminium and iron particles, respectively. The optical microscopy observations show that the iron powder is characterized by low porosity, as shown in Figure 4. The powders since the manufacturing unitl the moment of usage were stored under a protective argon atmosphere and, by that reason, were considered to possess a low degree of oxidation. X-ray phase analysis was also carried out for both powders; this analysis confirms that the purchased powders are actually iron and aluminum, and no traces of oxides were detected.

## 4. Experimental Procedure

The first step of the experiment was the preparation of powder mixtures from pure iron and aluminum with suitable atomic compositions: Fe-6% at. Al, Fe-28% at. Al, Fe-36% at. Al, and Fe-50% at. Al. The powder mixtures were prepared in an argon-filled container and mixed in the turbular mixer for 1hto prevent the oxidation of the powders. The samples were deposited on substrates with the same chemical composition as-deposited material but produced by sintering under load [77,78]. The substrates were manufactured in such a way to possibly lower the differences in the thermal expansion coefficients and improve the cohesion between the deposit and substrate.

**Figure 5:**
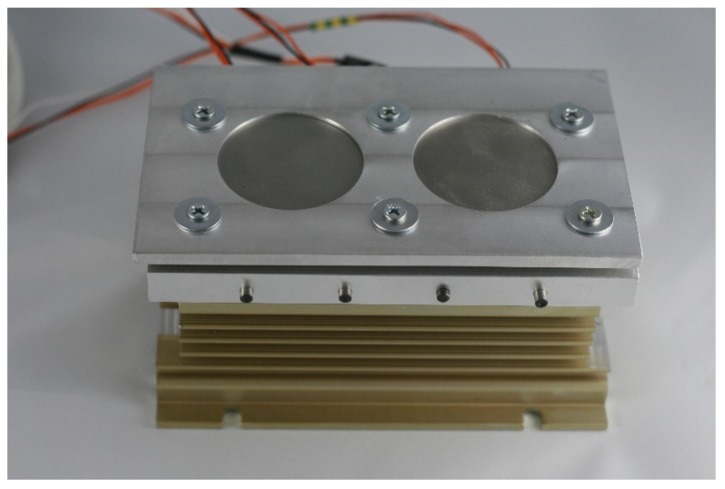
Heating table used during the LENS® process.

Subsequently, the substrates for LENS® were placed on a custom made heating table that was equipped with four heaters, where each substrate was connected to a thermocouple (Figure 5). The function of the table was to increase the substrate temperature to minimize the effects of the rapid change in temperature that were experienced in the heating-cooling cycle during the LENS® process. The heating table temperature was regulated by PID controller and attached to a XY platform of the device via a radiator. The temperature was set always before the deposition start and when reached, kept constant until the end of the process. The heating plate was made of aluminium alloy to provide a high homogeneity of the temperature filed. 

The samples were manufactured based on the CAD model, as 10 mm × 10 mm × 2mm plates. During the LENS® process, 12 samples were manufactured: four Fe6Al samples, five Fe28Al samples, two Fe36Al samples, and one Fe50Al sample. Table 1 provides the process parameters for each sample.

## 5. Results

### 5.1. Chemical Composition

**Figure 6:**
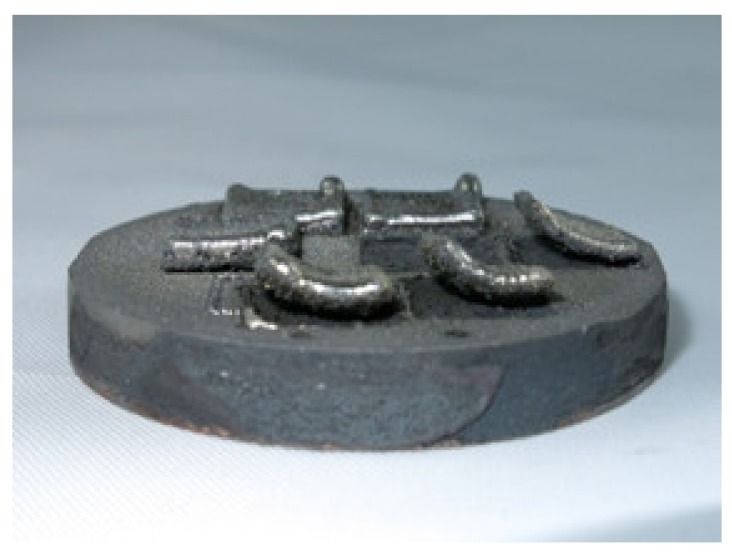
Fe6Al LENS® samples on a sintered Fe-Al substrate.

During the Fe6Al samples’ preparation, in each case, severe delamination occurred, and the deposited layers detached from the substrate (Figure 6). Consequently, only the chemical composition was examined for this group of samples(Table 2); no further studies were conducted and samples were considered to be unsuccessfully deposited. The chemical composition analyses confirmed the compatibility of the chemical composition with the assumed composition. 

Table 2 shows the chemical composition of each sample after LENS® manufacturing. Analysis of the results revealed that the LENS® technology allows for the manufacturing of intermetallics from elemental powders (Fe and Al). The chemical composition in each case was found to be closed to the designed one, proving that the chemical composition of the alloy can be easily tailored.

### 5.2. Microanalysis

**Figure 7:**
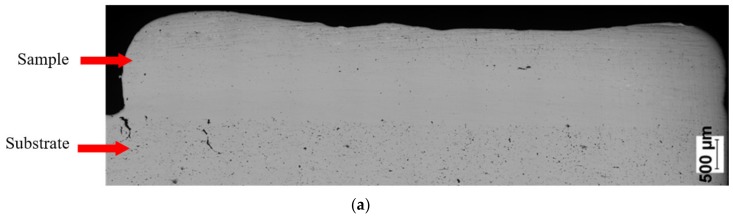

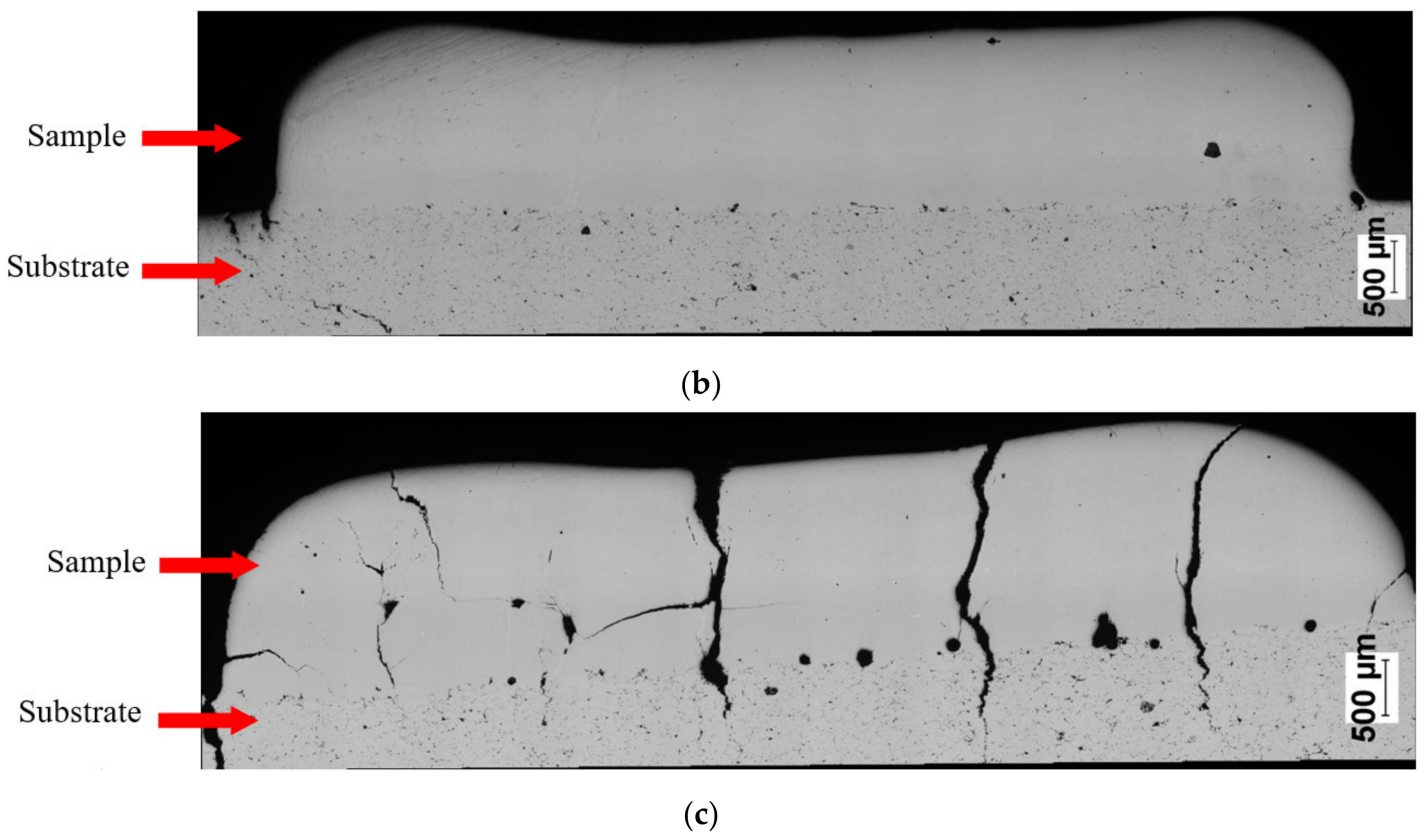
Optical microscope images of cross-sectionsof manufactured samples: (**a**) Fe28Al, (**b**) Fe36 Al, and (**c**) Fe50Al.

SEM observations revealed that the Fe28Al and Fe36Al samples are characterized by low porosity, and visible cracks are mainly in the interface or the bulk of the substrate (Figure 7a,b). The Fe50Al sample mainly has porosity at the substrate/layer interface (Figure 7c). The sample is strongly cracked, which is probably due to the thermal stresses that are generated during the LENS® process and well known low ductility of the FeAl alloys containing a large amount of aluminum. In addition, intermetallics with approximately 50 at.%Al have a tendency to possess the thermal vacancies [79,80], which also increases the brittleness of the product.

### 5.3. X-ray Diffraction Phase Analysis

**Figure 8:**
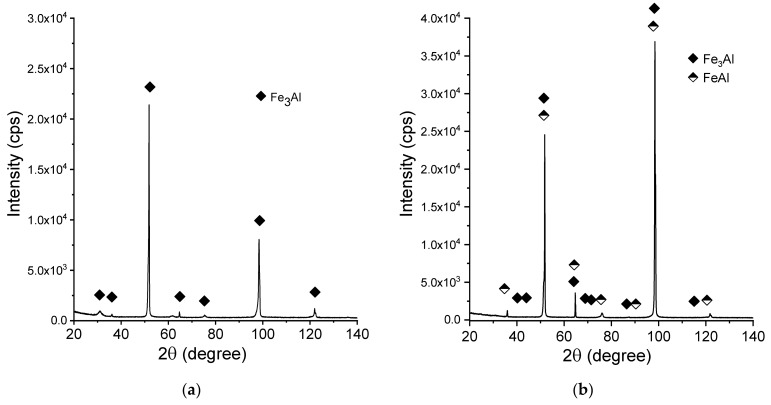

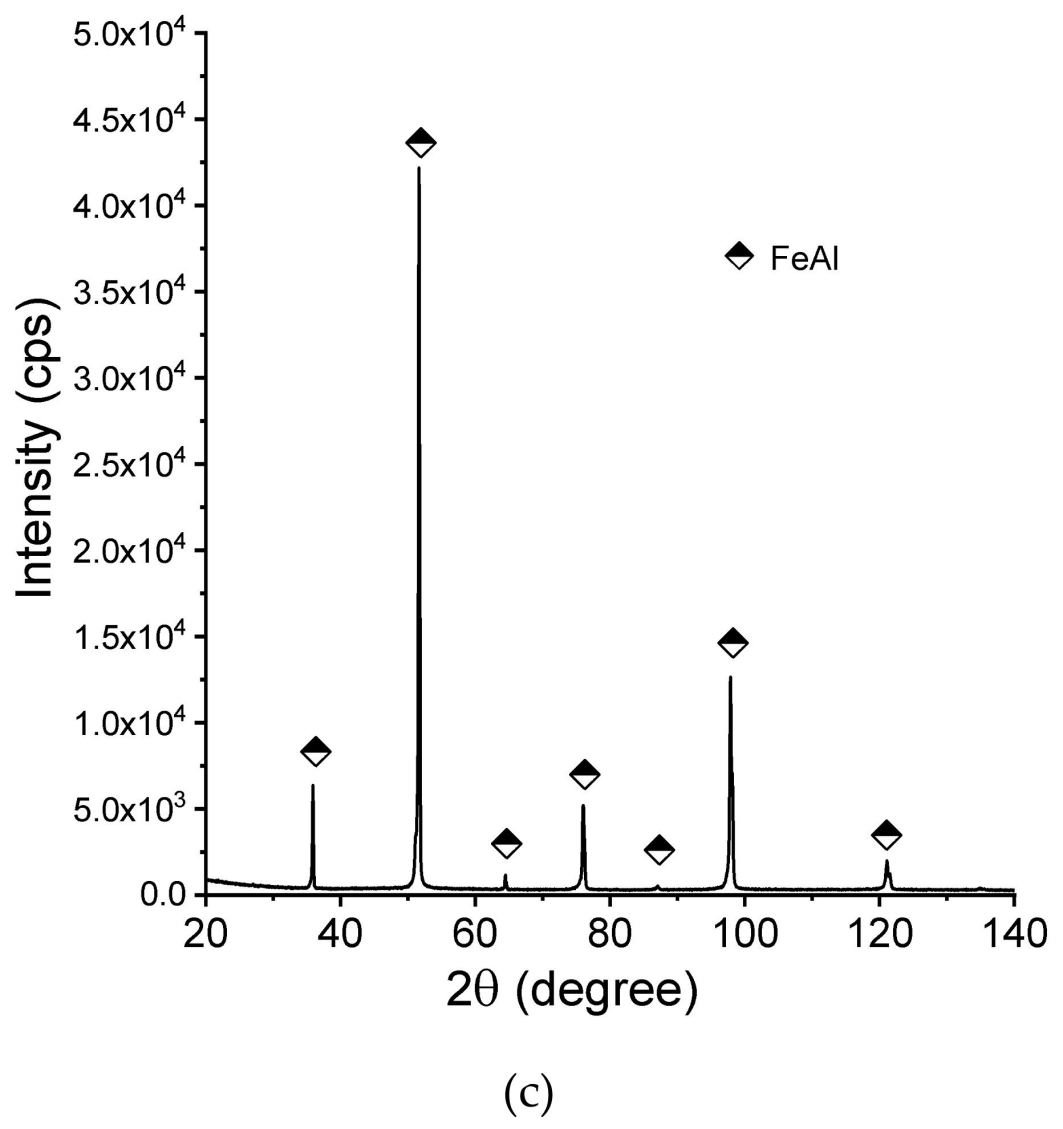
X-ray diffraction patterns (XRD)patterns of the manufactured samples:(**a**) Fe28Al, (**b**) Fe36Al, and (**c**) Fe50Al.

Figure 8 shows the X-ray diffraction patterns of the manufactured alloy. Sample with 28 % Al (at. %) was found to possess single-phase structure Fe_3_Al (D03) being the only constituent (Figure 8a). This is in accordance with what one can expect from this alloy based on its chemical composition. Introducing more aluminum to the alloy (36%) causes the two-phase structure to be obtained (Figure 8b), which is again corresponding well with what we can expect based on the well-known phase diagrams. Single-phase FeAlwas confirmed to exist in the sample with close to 50:50 % (Fe:Al) composition. No chemical composition segregation was observed. The differences in peaks relative intensities were observed for a single-phase Fe_3_Al sample and for a two-phase composition, which is related to the occurrence of texture in these samples.

### 5.4. Microhardness Test

**Figure 9:**
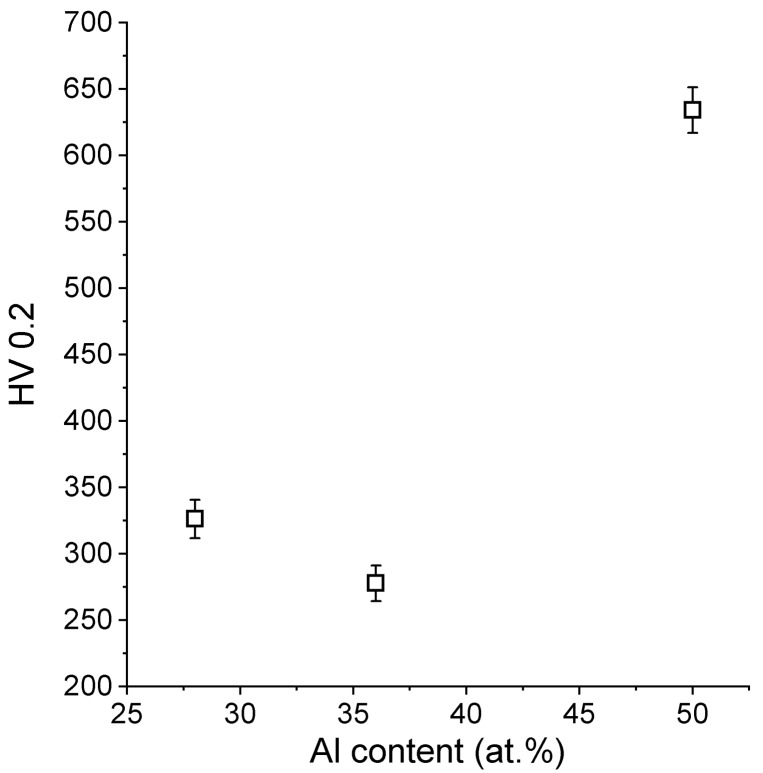
Dependence of the obtained microhardness results on the aluminum content.

Figure 9 gathers the results from the microhardness examinations of the LENS® samples. The obtained results did not show a specific linear correlation between the aluminum content and the obtained microhardness results. Normally, we would expect the hardness to increase with an increase of the aluminum content, knowing that the sample containing 36% of the aluminum is a mixture of two phases (Fe_3_Al and FeAl); however, this kind of behavior was already presented for FeAl by Frutoset al. [81]. It can be assumed that this behavior might also be caused by specific conditions of the LENS process (far from equilibrium-rapid cooling), which has already been noticed in previous works [45,82]. Similar experiments that were conducted by Shishkovsky et al. [72] have shown the hardness results more or less in the similar range, however it must be noted that they produced gradient samples and used aluminum alloy powder instead of pure aluminum, so the results are not easy to be compared.

## 6. Conclusions

Based on the obtained results, the following simple conclusions can be drawn:

1. The LENS technique can be used as an alternative approach to conventional alloying methods for the direct fabrication of Fe-Al alloys of the desired chemical and phase composition.
2. The application of mixtures of elemental powders (as a feedstock) allows the for production of alloys with desired chemical and phase compositions.
3. Thermal expansion during the process, as well as limited heat dissipation, results in cracks formation and delamination of the samples in some cases, despite the same chemical composition of the substrate and deposited layer.
4. The hardness of the manufactured alloys is very similar to the alloys that were produced by classical methods, and no direct linear correlation between the aluminum content and the hardness was found.

## Figures and Tables

**Table 1:** Process parameters of the LENS samples.

Sample No.	Powder	Laser Power (W)	Feedrate (mm/s)	Powder Output (Rpm)	SubstrateTemperature (°C)
6.1	Mixture (94at.%Fe+6at.% Al)	200	5	4	200
6.2		200	2.5	2	200
6.3		250	7.6	6	200
6.4		200	7.6	6	200
28.1	Mixture (72at.%Fe+28at.% Al)	200	5	4	200
28.2		200	7.5	6	200
28.3		250	10	8	200
28.4		250	12.5	10	200
28.5		200	12.5	10	200
36.1	Mixture (64at.%Fe+36at.% Al)	200	5	4	200
36.2		200	5	6	200
50.1	Mixture (50at.%Fe+50at.% Al)	200	5	6	200

**Table 2:** Chemical composition of manufactured samples.

Sample	Fe		Al	
	wt.%	at.%	wt.%	at.%
Fe6Al	96.9	93.9	3.1	6.1
Fe28Al	83.7	71.3	16.3	28.7
Fe36Al	80.7	66.8	19.3	33.2
Fe50Al	62.9	45.1	37.1	54.9
